# A new nomogram for individualized prediction of the probability of hemorrhagic transformation after intravenous thrombolysis for ischemic stroke patients

**DOI:** 10.1186/s12883-020-02002-w

**Published:** 2020-11-24

**Authors:** Yaya Wu, Hui Chen, Xueyun Liu, Xiuying Cai, Yan Kong, Hui Wang, Yun Zhou, Juehua Zhu, Lulu Zhang, Qi Fang, Tan Li

**Affiliations:** 1 Department of Neurology, The First Affiliated Hospital of Soochow University, Suzhou, Jiangsu Province China; 2 Department of Intensive Care Medicine, The First Affiliated Hospital of Soochow University, Suzhou, Jiangsu Province China; 3 Department of Neurology, The Second Hospital of Anhui Medical University, Hefei, Anhui Province China

**Keywords:** Ischemic stroke, Thrombolysis, Hemorrhagic transformation (HT), Nomogram

## Abstract

**Background:**

A reliable scoring tool to detect the risk of intracerebral hemorrhage (ICH) after intravenous thrombolysis for ischemic stroke is warranted. The present study was designed to develop and validate a new nomogram for individualized prediction of the probability of hemorrhagic transformation (HT) in patients treated with intravenous (IV) recombinant tissue plasminogen activator (rt-PA).

**Methods:**

We enrolled patients who suffered from acute ischemic stroke (AIS) with IV rt-PA treatment in our emergency green channel between August 2016 and July 2018. The main outcome was defined as any type of intracerebral hemorrhage according to the European Cooperative Acute Stroke Study II (ECASS II). All patients were randomly divided into two cohorts: the primary cohort and the validation cohort. On the basis of multivariate logistic model, the predictive nomogram was generated. The performance of the nomogram was evaluated by Harrell’s concordance index (C-index) and calibration plot.

**Results:**

A total of 194 patients with complete data were enrolled, of whom 131 comprised the primary cohort and 63 comprised the validation cohort, with HT rate 12.2, 9.5% respectively. The score of chronic disease scale (CDS), the global burden of cerebral small vascular disease (CSVD), National Institutes of Health Stroke Scale (NIHSS) score ≥ 13, and onset-to-treatment time (OTT) ≥ 180 were detected important determinants of ICH and included to construct the nomogram. The nomogram derived from the primary cohort for HT had C- Statistics of 0.9562 and the calibration plot revealed generally fit in predicting the risk of HT. Furthermore, we made a comparison between our new nomogram and several other risk-assessed scales for HT with receiver operating characteristic (ROC) curve analysis, and the results showed the nomogram model gave an area under curve of 0.9562 (95%CI, 0.9221–0.9904, *P* < 0.01) greater than HAT (Hemorrhage After Thrombolysis), SEDAN (blood Sugar, Early infarct and hyper Dense cerebral artery sign on non-contrast computed tomography, Age, and NIHSS) and SPAN-100 (Stroke Prognostication using Age and NIHSS) scores.

**Conclusions:**

This proposed nomogram based on the score of CDS, the global burden of CSVD, NIHSS score ≥ 13, and OTT ≥ 180 gives rise to a more accurate and more comprehensive prediction for HT in patients with ischemic stroke receiving IV rt-PA treatment.

**Supplementary Information:**

**Supplementary information** accompanies this paper at 10.1186/s12883-020-02002-w.

## Background

Intravenous thrombolysis (IVT) remains the standard treatment for patients with acute ischemic stroke (AIS) within 4.5 h after onset [1] and can improve clinical prognosis and reduce mortality [2]. However, it may cause serious complications, especially intracerebral hemorrhage transformation (HT), which leads to deteriorations of clinical neurological function and poor prognosis, thus limiting more widespread use of IVT. Therefore, a reliable scoring tool that can identify high risk of HT is essential.

Cerebral small vessel disease (CSVD) is a common finding among patients with AIS, which accounts for about 25% of all ischemic stroke patients [3]. Magnetic resonance (MR) is the gold standard imaging of CSVD, including white hyperintensities (WMHs), perivascular spaces (PVS), cerebral microbleeds (CMBs), lacunar infarction (LI) and brain atrophy. Previous studies showed that CSVD was related to hemorrhagic transformation and clinical prognosis after IVT [4, 5]. Recently, the concept of “total CSVD burden score”, which combines WMHs, PVS, CMBs and LI to indicate the joint effect of the CSVD subtypes, has drawn more and more attention. The total CSVD burden score can capture the overall effects of CSVD better than just one or two individual features separately. It has been applied in several fields including the cognitive function, stroke types and vascular risk factors [6–8]. In addition, the previous scoring systems applied in the past few years to predict the risk of intracerebral hemorrhage do not concern CSVD [9–14]. Nomogram, a graphical statistical instrument, has been generally performed in medical decision-making or other specialties by calculating the continous probability of a particular outcome for an individual patient [15–17].

In the present study, we aimed to develop and validate a new nomogram including the total burden score of CSVD and other related factors to predict the probability of HT for individual stroke patients with IV rt-PA treatment who would need more intensive monitoring and the extraordinary alterness.

## Methods

### Study population and data collection

The present study was a retrospective analysis of prospectively collected data from the Stroke Center of the First Hospital Affiliated of Soochow University between August 2016 and July 2018. All patients with acute cerebral infarction within 4.5 h from symptom onset were admitted and treated with IVT after the exclusion of the related contraindications. They underwent admission and finished MR-based imaging. We collected baseline and demographic characteristic, laboratory data and imaging information at admission and during hospitalization. The chronic disease scale (CDS) was rated from 0 to 3, which awarded one point for each of the following: hypertension (HTN), diabetes mellitus (DM) and atrial fibrillation (AF) [12]. Four putative strong predictors of HT (the score of CDS, the global burden of CSVD, baseline National Institutes of Health Stroke Scale (NIHSS) score, and onset-to-treatment time (OTT) for IVT) constitute the present nomogram. Patients who received endovascular mechanical thrombectomy or with unavailable clinical and imaging information were excluded from the analysis.All the data obtained in this study were evaluated separately by two professional physicians and corresponding thrombolytic hemorrhage risk scores were calculated according to previously published studies [9–13].

### Outcome

The HT was defined as any type of intracranial hemorrhage (ICH) according to ECASS II [18], which could be seen on non-contrast computed tomography (NCCT) of head and usually accurs within 12–36 h after IVT [19]. The types of HT included that: HI1 was defined as small petechiae along the margins of the infarct; HI2, as confluent petechiae within the infarcted area but no space-occupying effect; PH1, as blood clots in ≤30% of the infarcted area with some slight space-occupying effect; and PH2, as blood clots in > 30% of the infarcted area with a substantial space-occupying effect [18].

### The assessment of CSVD

The structure of magnetic resonance imaging (MRI) involved in the study included diffusion-weighted imaging (DWI), 3d-TOF-MRA, FLAIR, T2-weighted, T1-weighted, and gradient echo/T2*/ susceptibility weighted sequences.

The total CSVD burden score consisted of the following four imaging markers, including: (1) WMHs: WMHs were graded according to Fazekas’ scale [20]. One point was awarded as either confluent deep WMH (Fazekas score 2 or 3) or irregular periventricular WMH extending into the deep white matter (Fazekas score 3); (2) PVS: PVS were defined as small (< 3 mm) round or linear hyperintensities on T2 images in the basal ganglia or centrum semiovale. Presence of PVS would be counted if there were moderate to severe (Semiquantitative grade 2–4) [21] PVS in the basal ganglia (1 point if present). (3) CMBs or LI: CMBs were defined as small (< 5 mm), homogeneous, round foci of low signal intensity on gradient echo images in cerebellum, brainstem, basal ganglia, white matter, or cortico-subcortical junction [22]. Lacunes infarctions (LI) were defined as rounded or ovoid lesions, > 3 and < 20-mm diameter, in the basal ganglia, internal capsule, centrum semiovale, or brainstem, of CSF signal intensity on T2 and FLAIR, generally with a hyperintense rim on FLAIR, and no increased signal on DWI. One point was awarded if there is one or more lacunes or any CMB. Finally, we rated the total MRI burden of CSVD on an ordinal scale from 0 to 4 [23–25].

### Statistical analysis

Included patients were randomly assigned into two cohorts (7:3): the primary cohort and the validation cohort. Models were developed from the primary cohort and evaluated in the validation cohort. Continuous variables were compared using the Mann-Whitney U test for non-normally distributed variables or Student’s *t*-test for normally distributed variables. Differences between proportions were assessed by Fisher’s exact test or Chi-square test, where appropriate. Continuous variables were reported as the mean ± SD or median (interquartile range), and categorical variables were described by constituent ratio. The SPSS 22.0 was used for statistical analysis of baseline data.

To identify the independent predictors of HT, a multivariable logistic regression model was performed and the variables with a probability value < 0.05 were included in the univariate analysis. The optimal cut-off for independent parameters was determined using receiver operating characteristic (ROC) curve analysis with the highest value of Youden index, which was defined as specifity+sensitivy-1. After determining the optimal cut-off value, they were converted into dichotomous variables: lower than the cut-off value, equal to the cut-off value or higher than the cut-off value.

The nomogram including all the parameters above was constructed by R version 3.6.0. The nomogram was created by assigning a graphic preliminary score to each of the predictors with a point ranging from 0 to 100, which was then summed to generate a total score, finally converted to the logit and then to an individual probability (from 0 to 100%) of ICH. The performance of the nomogram was evaluated by Harrell’s concordance index (C-index) and the calibration plot [26]. C-index > 0.7 generally reflects a well-fitted feature of the predictive model. The overall predictive and discriminative performance of the nomogram for predicting the probability of ICH both in primary and validation cohort were compared with HAT(Hemorrhage After Thrombolysis), SEDAN (blood Sugar, Early infarct and hyper Dense cerebral artery sign on NCCT, Age, and NIHSS), SPAN-100(Stroke Prognostication using Age and NIHSS), THRIVE(Totaled Health Risks in Vascular Events) and GRASPS(Glucose, Race, Age, Sex, Systolic blood Pressure, and Severity of stroke) scores by using the estimate of the area under the receiver operating characteristic curve (AUC-ROC). The Calibration curve was performed with 1000 bootstrapped resamples for internal validation and was used to analyze the agreement between nomogram and actual observation. The packages of RMS and Hmisc were involved in this process.

## Results

### Clinical features and baseline characteristics

**Fig. 1:**
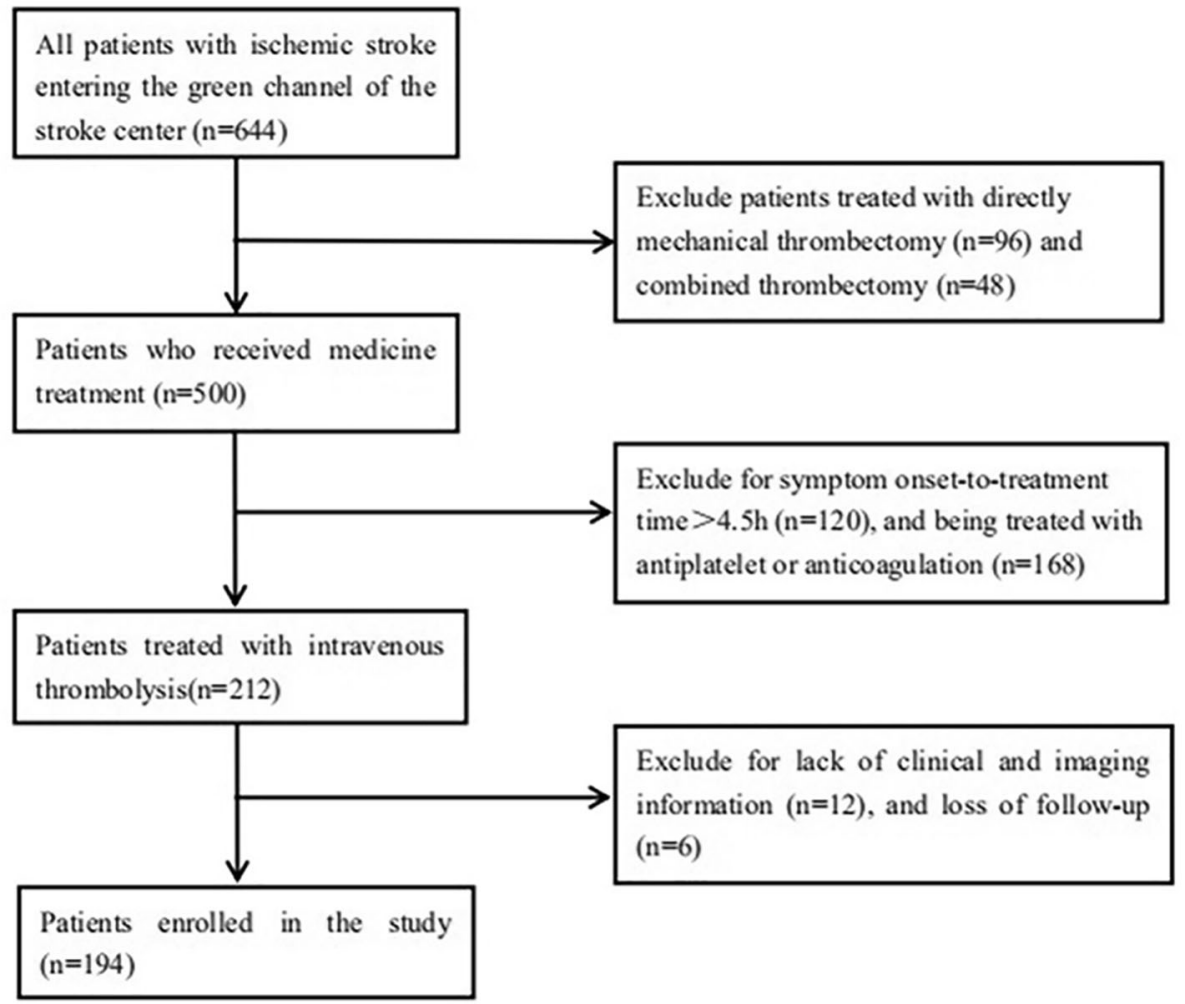
Flow diagram of included and excluded patients

The flow diagram of patient inclusion and exclusion is shown in Fig. 1. A total of 194 patients were included in the study for generating the nomogram. The baseline characteristics of patients in the primary and validation cohorts are shown in the Table 1. Of the 131 individuals comprising the primary cohort, the median age was 67 (59–74) years, and 74.0% were male, including 16 (12.2%) HT patients. The validation cohort consisted of 39 men (61.9%) and 24 women (38.1%) with a median age of 70 (62–78) years, and the overall proportion of HT was 6 (9.5%). The difference between the two cohorts was not statistically significant (all P>0.05).

**Table 1:** Baseline characteristics of the primary cohort and the validation cohort

Baseline	Primary cohort	Validation cohort	Test	*P* value
	(*n* = 131)	(*n* = 63)	statistic	
Age	67(59–74)	70(62–78)	− 1.441^a^	0.149
Sex, male	97(74.0)	39(61.9)	2.992	0.084
Admission SBP mmHg	152 ± 21	153 ± 21	−0.260^b^	0.796
Admission DBP mmHg	86(78–98)	85(78–92)	−0.378^a^	0.705
Admission glucose	5.7(4.9--6.8)	6.1(5.2--7.6)	−1.419^a^	0.156
Stroke	24(18.3)	6 (9.5)	2.518	0.113
Smoke	46(35.1)	19(30.2)	0.469	0.493
Dyslipidemia	20(15.3)	11(17.5)	0.152	0.696
Antiplatelets/anticoagulation	20(15.3)	9(14.3)	0.032	0.858
Hypertension	92(70.2)	40(63.5)	0.888	0.346
Diabetes	26(19.8)	18(28.6)	1.846	0.174
Atrial fibrillation	30 (22.9)	21(33.3)	2.389	0.122
CDS score			4.008	0.253
0	31(23.7)	14(22.2)		
1	60(45.8)	22(34.9)		
2	32(24.4)	24(38.1)		
3	8(6.1)	3(4.8)		
NIHSS score	6(3–11)	6(3–12)	−0.219^a^	0.827
OTT for thrombolysis min	160(120–200)	183(130–210)	−1.721^a^	0.085
Platelet *10^9 /L	181(152–215)	185(147–224)	0.198^a^	0.843
LDL mmol/L	2.58 ± 0.77	2.51 ± 0.62	0.626^b^	0.532
Creatinine umol/L	70.4(59.9–80.0)	69.9(55.4–84.0)	−0.868^a^	0.385
CRP mg/L	3.7(1.7–13.3)	4.6(1.3–10.5)	−0.582^a^	0.561
Uric acid mmol/L	300.2(241.0–351.3)	290.4(248.8–351.3)	−0.164^a^	0.870
CSVD score			0.575	0.966
0	23(17.6)	11(17.5)		
1	46(35.1)	24(38.1)		
2	39(29.8)	16(25.4)		
3	18(13.7)	10(15.9)		
4	5(3.8)	2(3.2)		
HT after thrombolysis	16(12.2)	6 (9.5)	0.306	0.580

*Abbreviations*: *SBP* systolic blood pressure, *DBP* diastolic blood pressure, *NIHSS* National Institutes of Health Stroke Scale, *OTT* onset-to-treatment time, *CDS* chronic disease scale, *LDL* low-density lipoprotein, *Hcy* homocysteine, *CRP* C-reactive protein, *CSVD* cerebral small vessel disease, *HT* hemorrhagic transformation

^a^represents the Z value obtained by Mann-Whitney U test, ^b^is t value by Student’s *t*-test, and the rest areλ^2^ value by Chi-square test

### The optimal cut-off value and risk factors for HT after thrombolysis

Considering the availability of variables, we determined the optimal cut-off values for predicting HT. The optimal cut-off value of NIHSS score was 13 to dichotomize patients. The optimal cut-off value of OTT for thrombolysis was 180 min. Univariable and multivariable logistic regression analysis showed that the score of CDS, the total burden score of CSVD, NIHSS score ≥ 13, and OTT ≥ 180 were important determinant of HT after thrombolysis (Table 2). Sensitivity and specificity of the above variables were provided in a data supplement (Table 1).

**Table 2:** Univariable model and Full multivariable model for the relationship between risk factors and HT after thrombolysis

	Univariable models		Full multivariable model	
	OR (95%CI)	*P* value	OR (95%CI)	*P* value
CDS score	6.32 (2.90–16.57)	<0.001	4.46 (1.87–13.09)	0.002
CSVD score	3.18 (1.82–6.12)	<0.001	2.94 (1.47–6.78)	0.004
NIHSS≥13	11.04 (3.53–36.49)	<0.001	24.53 (4.54–204.38)	0.001
OTT ≥ 180	3.01 (1.04–9.40)	0.046	7.77 (1.50–61.93)	0.026

*Abbreviations*: *CDS* chronic disease scale (hypertension, diabetes mellitus, atrial fibrillation), *CSVD* cerebral small vascular diseases, *NIHSS* National Institutes of Health Stroke Scale, *OTT* onset-to-treatment time for thrombolysis

### Prediction nomogram for HT after thrombolsis

**Fig. 2:**
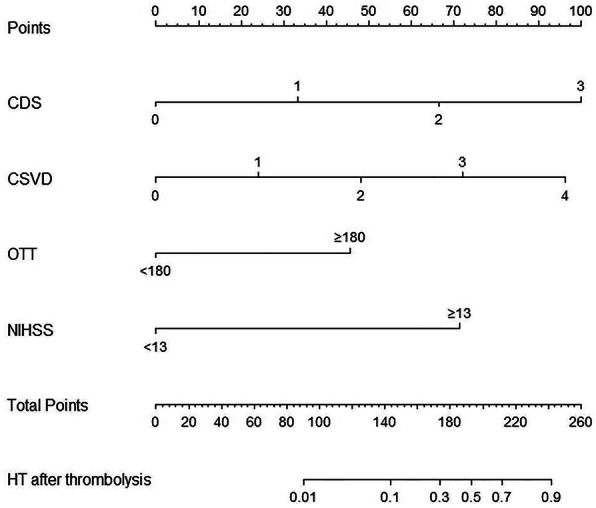
Nomogram predicting HT after IVT in patients with acute ischemic stroke. Abbreviations: CDS: chronic disease scale (hypertension, diabetes mellitus, atrial fibrillation); CSVD: cerebral small vascular diseases; NIHSS: National Institutes of Health Stroke Scale; OTT: onset-to-treatment time for thrombolysis

**Fig. 3:**
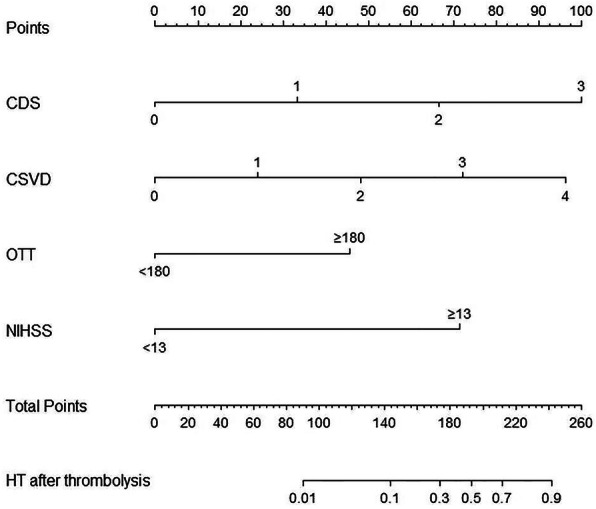
An example of how to use the nomogram to predict chance of HT after thrombolysis. In this example, we calculated the predicted probability of HT in a patient with hypertension, a NIHSS score of 5, the total CSVD burden score of 3, and the OTT time greater than 180 min. Points are assigned for each feature: 33 for the CDS score of 1, 72 for the total CSVD burden score of 3, 46 for OTT time greater than 180 min, and 0 for NIHSS score of 5. The total of 151 points corresponds to a nearly 13.7% chance of HT after thrombolysis for this patient.The formula for calculating probability of HT is provided in the data supplement. Abbreviations: CDS: chronic disease scale (hypertension, diabetes mellitus, atrial fibrillation); CSVD: cerebral small vascular diseases; NIHSS: National Institutes of Health Stroke Scale; OTT: onset-to-treatment time for thrombolysis

The significant risk factors above were used to construct the nomogram (Fig. 2). The nomogram was generated by assigning a graphic preliminary score to each of the 4 predictors with a point ranging from 0 to 100, which was then summed to generate the total score, finally converted into an individual probability of HT after thrombolysis (from 0 to 100%). To use the nomogram, you should identify the patient’s value for each predictive variable firstly, then draw a straight line upwards from each predictive value to the top point reference line and sum the points from each predictor, and finally find the location of the sum on the total points reference line and draw a straight line from total points line down to the bottom probability line to get the patient’s likelihood of HT. The applicability of the nomogram can be illustrated through a clinical example (Fig. 3). The specific parameters of the nomogram model and the formula for calculating probability of HT are provided in the data supplement.

### The discrimination and calibration performance of the model

The discriminatory ability of our model was assessed by using C-Statistics for HT prediction. The nomogram derived from the primary cohort for HT had C- Statistics of 0.9562 (95%CI, 0.9221–0.9904), and the discriminatory ability in the validation cohort was 0.9854 (95% CI, 0.9609–1.000).

**Fig. 4:**
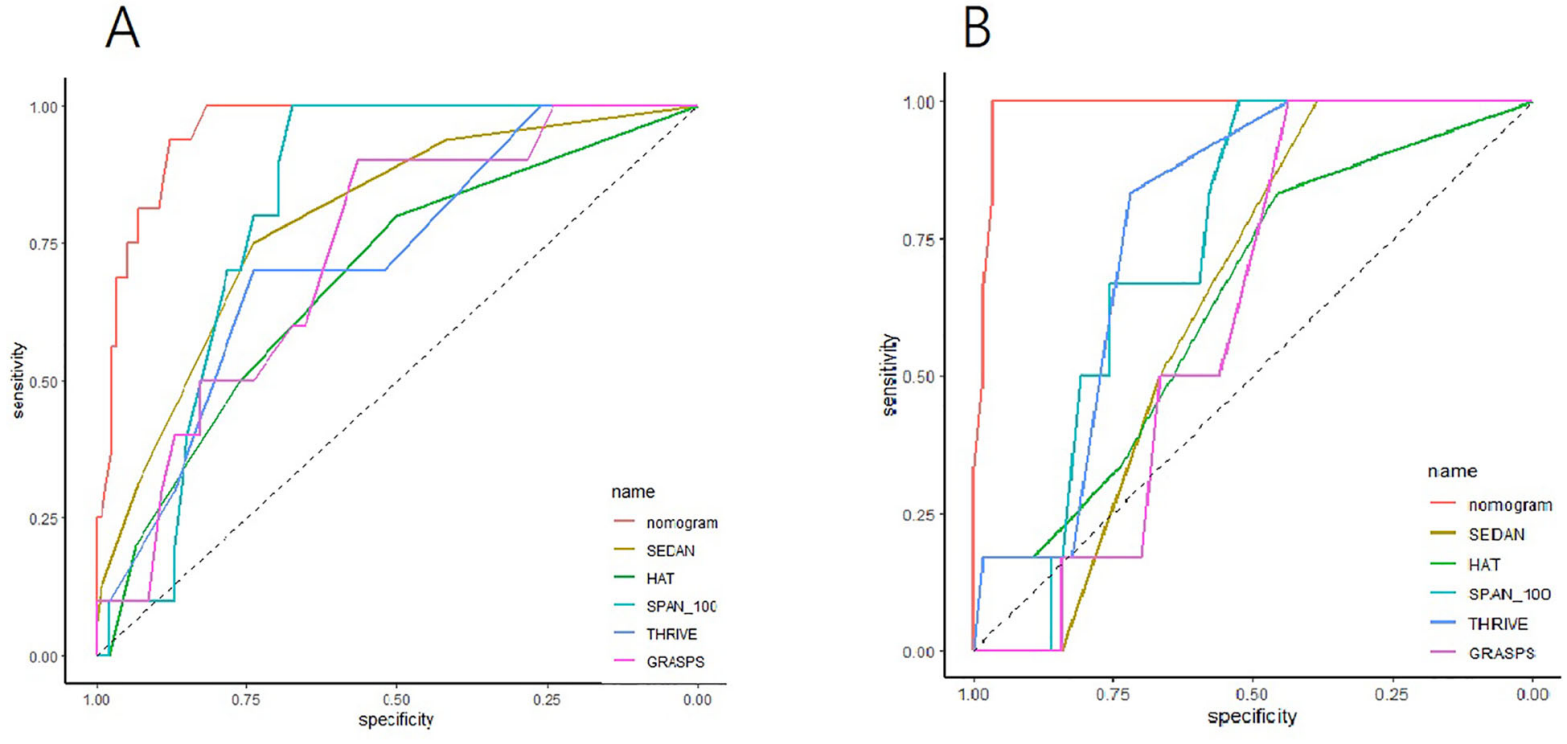
Receiver operating characteristic (ROC) curve analysis for the nomogram, HAT, SEDAN, SPAN-100, THRIVE and GRASPS in the primary cohort (**a**) and validation cohort (**b**). Abbreviations: HAT(Hemorrhage After Thrombolysis), SEDAN (blood Sugar, Early infarct and hyper Dense cerebral artery sign on NCCT, Age, and NIHSS), SPAN-100(Stroke Prognostication using Age and NIHSS), THRIVE(Totaled Health Risks in Vascular Events) and GRASPS(Glucose, Race, Age, Sex, Systolic blood Pressure, and Severity of stroke)

We further compared our nomogram with HAT, SEDAN, SPAN-100 and GRASPS by using the estimate of AUC-ROC. The results showed that the new model was superior to HAT, SEDAN and SPAN-100 (all *P* < 0.001) in the primary cohort, suggesting that our model provided statistically significant value for predicting HT after thrombolysis (Table 3, Fig. 4). The Table 2 (in the data supplement) showed sensitivity and specificity of nomogram and above scoring systems in the two cohorts respectively.

**Table 3:** The comparison of AUC-ROC of the nomogram, HAT, SEDAN, SPAN-100, THRIVE and GRASPS for prognosis in the primary cohort and validation cohort

Scales	Primary cohort			Validation cohort		
	AUC-ROC	95%CI	*P* value	AUC-ROC	95%CI	*P* value
Nomogram	0.9562	0.9221–0.9904		0.9854	0.9609–1.000	
HAT	0.8082	0.7099–0.9064	<0.01	0.6374	0.4183–0.8566	<0.01
SEDAN	0.7916	0.6807–0.9025	<0.01	0.6404	0.4992–0.7815	<0.01
SPAN-100	0.8174	0.7069–0.9279	<0.01	0.7339	0.5923–0.8755	<0.01
THRIVE	0.8641	0.7604–0.9678	0.0567	0.7763	0.6423–0.9103	<0.01
GRASPS	0.8239	0.7128–0.9350	0.0148	0.6170	0.4604–0.7735	<0.01

*Abbreviations*: *HAT* Hemorrhage After Thrombolysis, *SEDAN* blood Sugar, Early infarct and hyper Dense cerebral artery sign on NCCT, Age, and NIHSS, *SPAN-100* Stroke Prognostication using Age and NIHSS, *THRIVE* Totaled Health Risks in Vascular Events, and *GRASPS* Glucose, Race, Age, Sex, Systolic blood Pressure, and Severity of stroke, *AUC-ROC* area under the receiver operating characteristic curve

**Fig. 5:**
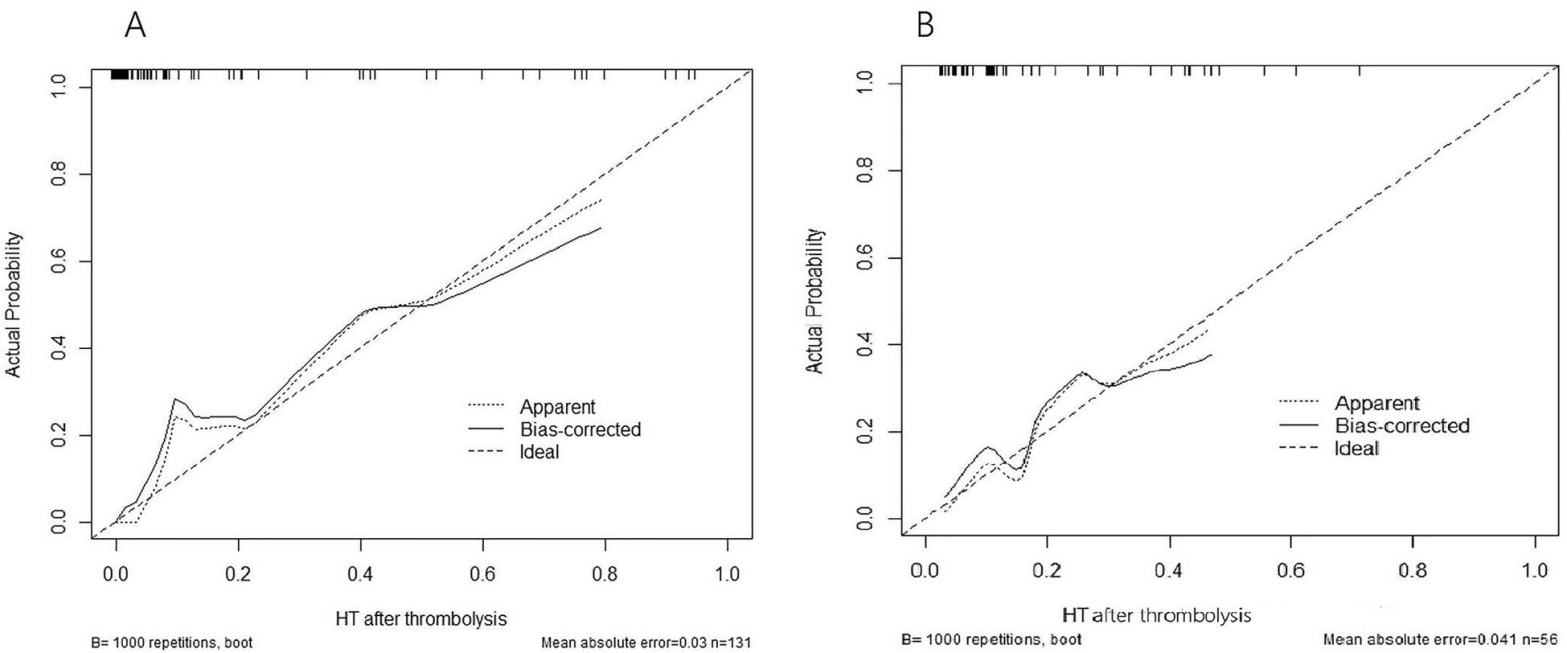
The calibration curves for predicting HT of patients receiving thrombolysis in the primary cohort (**a**) and validation cohort (**b**). Abbreviations: HAT(Hemorrhage After Thrombolysis), SEDAN (blood Sugar, Early infarct and hyper Dense cerebral artery sign on NCCT, Age, and NIHSS), SPAN-100(Stroke Prognostication using Age and NIHSS), THRIVE(Totaled Health Risks in Vascular Events) and GRASPS(Glucose, Race, Age, Sex, Systolic blood Pressure, and Severity of stroke)

The calibration curves for the probability of HT after thrombolysis was demonstrated in the Fig. 5. The x-axis represented the predicted HT resulted from the nomogram, and the y-axis exhibited actual HT. The calibration plot revealed general fit of the nomogram predicting the risk of HT both in the primary cohort and validation cohort.

## Discussion

We presented here a new nomogram based upon the score of CDS, the total burden score of CSVD, NIHSS score, and OTT to predict the probability of HT for ischemic stroke patients treated with IV rt-PA. The discriminatory and calibration capacity of nomogram model was then confirmed by the primary cohort and the validation cohort from the same data set, and was compared with HAT, SEDAN, SPAN-100, THRIVE and GRASPS scores by AUC-ROC.

Nomogram is generally performed as a statistical prognostic model with the ability to generate an individual probability of a clinical event by integrating diverse prognostic and determinant variables, which meet our desire for biologically and clinically integrated models and fulfill our drive towards personalised medicine [27]. CSVD is a common finding among patients with acute ischemic stroke, and its main manifestations on magnetic resonance are white matter hyperintensities, cerebral microbleeds, peripheral vascular space, lacunar infarction and cerebral atrophy. Recent studies have shown that white matter hyperintensities and cerebral microbleeds could increase the risk of hemorrhagic transformation after thrombolysis in stroke patients [4, 5]. Several scoring systems have been published for early prediction of ICH after thrombolysis in ischemic stroke patients [9–14]. Clearly, these scoring systems do not include any CSVD MRI markers. Besides, some recent studies have shown that the total CSVD burden score may better reflect the overall impact of CSVD on the cerebral hemisphere in a simple and practical way than considering one or two separate features [25]. Therefore, our study constructed a new nomogram model comprising the global burden of CSVD, the score of CDS, NIHSS score and OTT for thrombolysis. The new model presented good discriminatory ability with C- Statistics of 0.9562 (95%CI, 0.9221–0.9904). The model has acceptable calibration suggesting a general consistency between the predicted risk and the actual risk of HT. For patients with high probability of HT, we can timely strengthen the monitoring and treatment of blood glucose and blood pressure, arrange imaging review and avoid early use of antithrombotic drugs. The model could also be used to stratify patients in large studies such as testing new devices, thrombolytic or neuroprotective drugs. Besides, if relevant software is available on a computer or mobile device, our nomogram may be easily and quickly applicable to clinicians.

The important predictor for ICH included in the nomogram was the total CSVD burden score (OR = 2.94,95%CI: 1.47–6.78, *P* = 0.004). Total CSVD burden score was a new scale proposed in recent years for more accurate evaluation of all brain injuries from CSVD. Recently, some researchers have paid attention to the relationship between total CSVD burden score and thrombolysis. Arba F et.al showed that the total CSVD burden score was correlated with disability and functional dependence [28]. Another research that combined the results of two large cohort studies pionted out that patients with a higher total CSVD burden score had a significant increased risk of recurrent ischemic stroke and hemorrhagic transformation [29]. In consistence with the above results, our single-center study found that the total CVSD burden score was a reliable predictor for poor outcomes with a suggested CSVD score ≥ 2 [30]. We also found that the total CSVD burden score was closely related to the transformation of bleeding after thrombolysis.

NIHSS score was another well-known predictor of HT after IV rt-PA., and it was commonly included in the existing assessment scores for hemorrhagic risk after thrombolysis. NIHSS score 7 to 12 and ≥ 13 were the risk categories incorporated into the SITS score [14]. In SEDAN scale, the cutoff value of NIHSS was 10 point [11]. The risk categories of NIHSS score in GRASPS scale were 0 to 5, 6 to 10, 11 to 15, 16 to 20, and > 20 [13]. In our study, we determined that the initial NIHSS score ≥ 13 was independently associated with ICH after IV rt-PA (OR, 24.53; 95%CI, 4.54–204.38; *P* = 0.001), and attributed 71 scores in our model for individual prediction. The differences of the NIHSS cut-off values in the above scales might be due to the differences in study population and study methods. Therapeutic efficacy of rt-PA was time dependent and better outcomes could be expected when treatment was administered sooner rather than later within the therapeutic window. Hence, we included OTT in the model, an indicator of the quality of prehospital emergency medical service system and inhospital green channel for AIS. In consistence with Michael Mazya’s reseach [14], our study proved OTT ≥ 180 min as the distinction point for HT (OR, 7.77; 95CI,1.50–61.93; *P* = 0.026), and attributed a score of 46 points in our nomogram model. Meanwhile, our study confirmed that the score of CDS (HTN, DM, and AF) was a significant predictor for HT after thrombolysis (OR,4.46; 95% CI, 1.87–13.09; *P* = 0.002). The total score of CDS were 1, 2, and 3 that attributed a score of 33 points, 67 points, 100 points respectively.

To our knowledge, the present study was the first one of the HT assessment scales that included the total CSVD burden score. Besides, NIHSS score, the score of CDS, and OTT for thrombolysis were also included in our nomogram model to explore the risk of HT after thrombolysis. The most appealing aspect of our model was that it contained imaging biomarkers and used the global load score of CSVD to reflect overall brain damage. Moreover, we used discrimination and calibration to assess the performance of our model, and the results were satisfactory.

There are still some limitations in the present study. Firstly, it is a single-center retrospective analysis with a small sample. Although the discriminative performance of the model was good, an external validation in a completely different cohort was still warranted before the formal routine clinical practice. Besides, data on platelet count, international normalized ratio, and activated partial thromboplastin time values are not available in the model to assess their possible association with HT. Lastly, a few patients with severe symptomatic intracranial hemorrhage and subarachnoid hemorrhage who could not bear MR imaging were not included in our study, so this might lead to bias in case selection.

## Conclusions

We proposed and validated a nomogram model that included the score of CDS, the total burden of CSVD, NIHSS score and OTT for thrombolysis, which reliably calculated the probability of HT in AIS patients receiving intravenous rt-PA.

## Supplementary Information

**Additional file 1:** **Table S1.** Sensitivity and specificity of the variables concluded in nomogram. **Table S2.** Sensitivity and specificity of nomogram, HAT, SEDAN, SPAN-100, THRIVE and GRASPS in the primary cohort and validation cohort.

## Data Availability

The datasets generated during the current study are available from the corresponding author on reasonable request.

## Abbreviations

AF: Atrial fibrillation
AIS: Acute ischemic stroke
AUC-ROC: Area under the receiver operating characteristic curve
CDS: Chronic disease scale
CMBs: Cerebral microbleeds
CSVD: Cerebral small vascular disease
DM: Diabetes mellitus
DWI: Diffusion-weighted imaging
ECASS II: European Cooperative Acute Stroke Study II
GRASPS: Glucose, Race, Age, Sex, Systolic blood Pressure, and Severity of stroke
HAT: Hemorrhage After Thrombolysis
HT: Hemorrhagic transformation
HTN: Hypertension
ICH: Intracerebral hemorrhage
IV: Intravenous
IVT: Intravenous thrombolysis
LI: Lacunar infarction
MRI: Magnetic resonance imaging
NCCT: Non-contrast computed tomography
NIHSS: National Institutes of Health Stroke Scale
OTT: Onset-to-treatment time
PVS: Perivascular spaces
rt-PA: Recombinant tissue plasminogen activator
SD: Standard deviation
SEDAN: Blood Sugar, Early infarct and hyper Dense cerebral artery sign on non-contrast computed tomography, Age, and NIHSS
SPAN-100: Stroke Prognostication using Age and NIHSS
THRIVE: Totaled Health Risks in Vascular Events
WMHs: White hyperintensities
