# Supplementary material for: A new nomogram for individualized prediction of the probability of hemorrhagic transformation after intravenous thrombolysis for ischemic stroke patients
Source: BMC Neurol. 2020 Nov 24;20:426. doi: 10.1186/s12883-020-02002-w (PMC7685652; doi:10.1186/s12883-020-02002-w)
Supplement: Supplementary file 1 — Additional file 1: Table S1. Sensitivity and specificity of the variables concluded in nomogram. Table S2. Sensitivity and specificity of nomogram, HAT, SEDAN, SPAN-100, THRIVE and GRASPS in the primary cohort and validation cohort. [file 12883_2020_2002_MOESM1_ESM.docx]

**The specific parameters of the nomogram model and the formula for calculating probability of HT**

In the nomogram, the line labelled Points is used to calculate the points associated with each of the four variables. The subsequent lines (lines 2-5 in figure 2) are the risk factors used in the model. The value for each variable is located on these lines and a vertical line is drawn up to the Points line to find the points associated with each value. All of the points are totalled and the sum located on the Total points line. A vertical line is drawn down to locate the predicted log hazard of the model and the predicted chance of hemorrhagic transformation after intravenous thrombolysis.

The AIC of the model was 55.15, Cox & Snell R Square was 0.328 and Nagelkerke R Square was 0.626.

The formula for calculating probability of HT was: -4.87e-07 * points ^3 + 0.000278158 * points ^2 + -0.043405532 * points + 2.021943492.

**Table 1** **Sensitivity and specificity of the variables concluded in nomogram.**

| variables | AUC-ROC | Sensitivity | Specificity | Youden index |
| --- | --- | --- | --- | --- |
| CDS score | 0.844 | 68.2% | 88.4% | 0.566 |
| CSVD score | 0.816 | 54.5% | 83.7% | 0.382 |
| NIHSS≥13 | 0.691 | 86.4% | 72.1% | 0.585 |
| OTT≥180 | 0.654 | 72.7% | 58.1% | 0.308 |

Abbreviations: CDS: chronic disease scale (hypertension, diabetes mellitus, atrial fibrillation); CSVD: cerebral small vascular diseases; NIHSS: National Institutes of Health Stroke Scale; OTT: onset-to-treatment time for thrombolysis; AUC-ROC: area under the receiver operating characteristic curve.

**Table 2** **Sensitivity and specificity of nomogram ,HAT, SEDAN, SPAN-100, THRIVE and GRASPS in the primary cohort and validation cohort.**

| Scales | Primary cohort | | | validation cohort | | |
| --- | --- | --- | --- | --- | --- | --- |
|  | Sensitivity | Specificity | Youden index | Sensitivity | Specificity | Youden index |
| Nomogram | 95.5% | 87.2% | 0.827 | 100% | 96.5% | 0.965 |
| HAT | 93.8% | 55.7% | 0.495 | 83.3% | 45.6% | 0.289 |
| SEDAN | 75.0% | 73.9% | 0.489 | 100% | 50.0% | 0.500 |
| SPAN-100 | 75.0% | 77.4% | 0.524 | 100% | 52.6% | 0.526 |
| THRIVE | 75.0% | 87.8% | 0.628 | 83.3% | 71.9% | 0.552 |
| GRASPS | 81.3% | 75.7% | 0.570 | 100% | 43.9% | 0.439 |

Abbreviations: HAT( Hemorrhage After Thrombolysis), SEDAN (blood Sugar, Early infarct and hyper Dense cerebral artery sign on NCCT , Age, and NIHSS), SPAN-100(Stroke Prognostication using Age and NIHSS), THRIVE(Totaled Health Risks in Vascular Events) and GRASPS( Glucose, Race, Age, Sex, Systolic blood Pressure, and Severity of stroke); AUC-ROC: area under the receiver operating characteristic curve.
